# Shared and specific dynamics of brain activity and connectivity in amnestic and nonamnestic mild cognitive impairment

**DOI:** 10.1111/cns.13937

**Published:** 2022-08-17

**Authors:** Xiaomei Zhong, Ben Chen, Le Hou, Qiang Wang, Meiling Liu, Mingfeng Yang, Min Zhang, Huarong Zhou, Zhangying Wu, Si Zhang, Gaohong Lin, Yuping Ning

**Affiliations:** ^1^ Center for Geriatric Neuroscience The Affiliated Brain Hospital of Guangzhou Medical University, Memory Clinic Guangzhou Guangdong Province China; ^2^ Department of Neurology The Affiliated Brain Hospital of Guangzhou Medical University Guangzhou Guangdong Province China; ^3^ Department of Geriatric Psychiatry The Second People's Hospital of Dali Bai Autonomous Prefecture Dali Yunnan Province China; ^4^ The First School of Clinical Medicine, Southern Medical University Guangzhou Guangdong Province China; ^5^ Guangdong Engineering Technology Research Center for Translational Medicine of Mental Disorders Guangzhou China

**Keywords:** Alzheimer's disease, dynamic networks, functional connectivity, mild cognitive impairment, MRI, neuroimaging

## Abstract

**Aims:**

The present study aimed to compare temporal variability in the spontaneous fluctuations of activity and connectivity between amnestic MCI (aMCI) and nonamnestic MCI (naMCI), which enhances the understanding of their different pathophysiologies and provides targets for individualized intervention.

**Methods:**

Sixty‐five naMCI and 48 aMCI subjects and 75 healthy controls were recruited. A sliding window analysis was used to evaluate the dynamic amplitude of low‐frequency fluctuations (dALFF), dynamic regional homogeneity (dReHo), and dynamic functional connectivity (dFC). The caudal/rostral hippocampus was selected as the seeds for calculating dFC.

**Results:**

Both aMCI and naMCI exhibited abnormal dALFF, dReHo, and hippocampal dFC compared with healthy controls. Compared with individuals with naMCI, those with aMCI exhibited (1) higher dALFF variability in the right putamen, left Rolandic operculum, and right middle cingulum, (2) lower dReHo variability in the right superior parietal lobule, and (3) lower dFC variability between the hippocampus and other regions (left superior occipital gyrus, middle frontal gyrus, inferior cerebellum, precuneus, and right superior frontal gyrus). Additionally, variability in dALFF, dReHo, and hippocampal dFC exhibited different associations with cognitive scores in aMCI and naMCI patients, respectively. Finally, dReHo variability in the right superior parietal lobule and dFC variability between the right caudal hippocampus and left inferior cerebellum exhibited partially mediated effects on the different memory scores between people with aMCI and naMCI.

**Conclusion:**

The aMCI and naMCI patients exhibited shared and specific patterns of dynamic brain activity and connectivity. The dReHo of the superior parietal lobule and dFC of the hippocampus‐cerebellum contributed to the memory heterogeneity of MCI subtypes. Analyzing the temporal variability in the spontaneous fluctuations of brain activity and connectivity provided a new perspective for exploring the different pathophysiological mechanisms in MCI subtypes.

## Graphical abstract

The aMCI and naMCI patients exhibited shared and specific patterns of dynamic brain segregation and integration. The segregation of superior parietal lobule and integrations of hippocampus‐cerebellum contributed to the memory heterogeneity of MCI subtypes. These results may not only enhance the understanding of the different mechanisms between MCI subtypes but also provide more potential targets for their neuromodulation and prevent them from developing dementia.

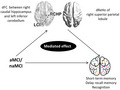

## 1 INTRODUCTION

Mild cognitive impairment (MCI) is considered a transitional stage between normal aging and dementia^1^ and can be divided into amnestic mild cognitive impairment (aMCI) and nonamnestic mild cognitive impairment (naMCI). MCI subtypes are not only theoretical but also underpinned by different pathophysiologies and disease trajectories;^2^ aMCI is more likely to develop into Alzheimer's disease,^3^, ^4^ and naMCI is more related to other kinds of dementia, such as vascular dementia or dementia with Lewy bodies.^5^ Additionally, MCI subtypes differ in aspects of susceptible genes, cardiovascular risk factors, progression courses,^6^, ^7^ and patterns of brain abnormalities.^8^ Therefore, a deeper understanding of the differences in MCI subtypes will not only contribute to the prediction of dementia type but also provide more therapeutic strategies for preventing the development of dementia.

The different patterns of brain abnormalities between aMCI and naMCI patients have been repeatedly revealed by magnetic resonance imaging (MRI) research. For structural MRI, studies have demonstrated that there are significant differences in the morphology and integrity of gray matter^8^, ^9^, ^10^ and white matter between aMCI and naMCI patients.^11^, ^12^ Additionally, functional MRI studies suggested that aMCI and naMCI patients exhibited differences in activity and connectivity: (1) aMCI patients exhibited a decreased amplitude of low‐frequency fluctuations (ALFF) in the superior temporal gyrus, insula, precentral gyrus, lingual gyrus, and superior frontal gyrus compared with naMCI groups and controls;^13^, ^14^ (2) aMCI patients but not naMCI patients exhibited decreased regional homogeneity (ReHo) in the anterior cingulate gyrus compared with controls;^14^ (3) compared with controls, aMCI patients and naMCI patients exhibited a different pattern of functional connectivity (FC) between the hippocampus and posterior cingulate cortex^15^ and FC within the default mode network;^16^ and (4) aMCI patients and naMCI patients exhibited different patterns of activation in temporal‐parietal regions during memory recognition compared with controls.^17^ Moreover, aMCI and naMCI patients exhibited opposite associations between Theory of mind performance and FC between the bilateral temporal pole and the left lateral temporal cortex.^18^

All the mentioned studies mainly focus on the static aspect of functional abnormalities, which assume that brain activity and connectivity are static over a whole resting‐state functional MRI scan. However, evidence from both task‐based fMRI studies and animal electrophysiology demonstrates that functional activity and connectivity may exhibit dynamic changes within time scales of seconds to minutes.^19^ Additionally, spontaneous fluctuations in brain activity and connectivity have long been recorded in electrophysiological recordings of single cells, local fields, and surface electroencephalograms.^20^ Therefore, important information can be missed when using average functional activity connectivity as the analytical method. Compared with stationary analyses, dynamic analyses facilitate the observation of details that are averaged out in stationary analyses and may offer greater insight into the fundamental mechanisms of activity and connectivity. Additionally, dynamic analyses enable the capture of spontaneously reoccurring patterns of activity and connectivity, which is essential for understanding the temporal variability in the intrinsic organization of the brain.^21^ By using the dynamic sliding window method throughout the scanning procedure, the dynamic characteristics of brain function, such as dynamic ALFF (dALFF), dynamic ReHo (dReHo), and dynamic FC (dFC), can be captured effectively.^22^, ^23^ Several researchers have successfully applied dynamic analyses to neuropsychiatric diseases, such as AD,^24^ Parkinson's disease,^25^ bipolar disease, depression,^26^, ^27^ and schizophrenia,^28^ which provide a novel understanding of their pathophysiologies.

For MCI individuals, studies suggested that they exhibited different patterns of dALFF compared with healthy controls in the working memory state,^29^ and the dALFF in the left calcarine cortex was higher in MCI patients than in AD patients.^30^ Additionally, a combination of dFC improved the diagnostic performance of MCI from healthy controls.^31^, ^32^ This evidence suggests that dynamic analyses enable the capture of spontaneously reoccurring patterns of activity and connectivity in patients with MCI and AD, which provide better knowledge of the pathophysiology of MCI. Nevertheless, the different patterns of dynamic brain function between aMCI and naMCI patients have not yet been investigated. Exploring the different dynamic characteristics of brain function between aMCI and naMCI patients may not only enhance the understanding of the different mechanisms between MCI subtypes but also provide more potential targets for their neuromodulation and prevent them from developing dementia.

Therefore, a sliding window analysis was performed in the present study to compute dALFF, dReHo, and dFC to characterize the temporal variability in the spontaneous fluctuations of activity and connectivity in MCI subtypes in comparison with a cognitively healthy group. We hypothesized that aMCI and naMCI patients would show both shared and specific patterns of abnormal dynamic brain activity and connectivity and that the difference in dynamic characteristics would be associated with their different patterns of cognitive impairment.

## 2 METHODS

### 2.1 Subjects

A total of 188 subjects were recruited from the Affiliated Brain Hospital of Guangzhou Medical University and the community in Guangzhou. All subjects or their legal guardians provided signed informed consent to participate in the study. The study was conducted in accordance with the Declaration of Helsinki and approved by the ethics committees of the Affiliated Brain Hospital of Guangzhou Medical University (Guangzhou Huiai Hospital) (2014, 078).

The inclusion criteria for patients with naMCI were as follows: (1) normal overall cognitive function as evidenced by a Clinical Dementia Rating (CDR) ≤0.5, activities of daily living (ADL) score = 14, Mini‐Mental State Examination (MMSE) score higher than the adjusted scores (illiterate ≥17 points, primary school ≥20 points, and above middle school ≥24), Hamilton Depression Rating Scale (HAM‐D) score ≤7; and (2) objective impairment in at least one cognitive domain except memory function, including language function, visuospatial skill, executive function, and attention. The inclusion criteria for patients with aMCI were as follows: (1) patients complained of memory impairment for at least 3 months or relatives confirmed that the memory impairment had lasted for more than 3 months; (2) objective memory performance documented by an auditory verbal learning test delayed recall score within ≤4; (3) normal overall cognitive function as described for naMCI; and (4) no dementia.^1^, ^15^, ^16^ The inclusion criteria for HCs were (1) no memory complaints; (2) normal cognitive performance; and (3) CDR = 0.

The exclusion criteria for all subjects were as follows: (1) psychiatric illness (such as schizophrenia and bipolar disorders); (2) physical disease that may cause cognitive or mental abnormalities (such as hypothyroidism and anemia); (3) a major neurological disease (such as Parkinson's disease and stroke); (4) claustrophobia or metal implants that precluded MRI scans; and (5) present or previous psychotic symptoms.

### 2.2 Neuropsychological assessments

After undergoing standard clinical assessments, participants were interviewed by neuropsychologists to assess global cognitive function using the Mini‐Mental State Examination (MMSE) (>24 as normal),^33^ following a battery of neuropsychological tests to assess performance in 5 cognitive domains: memory (auditory verbal learning test (AVLT) (N5 > 4 as normal));^34^ language (Boston Naming Test (BNT) (>22 as normal),^35^ verbal fluency test (VFT) (>10 as normal));^36^ executive function (Stroop Color and Word Test (Stroop) C (<113s as normal),^37^ trail‐making test Part B (TMT B) (<200s as normal));^38^ attention (Symbol Digit Modalities Test (SDMT) (>28 as normal),^39^ digit span test (DST) (> as normal));^40^ and visuospatial skills (clock drawing test 4 (CDT4) (=4 as normal),^41^ Rey‐Osterrieth complex figure test (ROCF) (>30 as normal)).^42^ The AVLT N1‐3 was defined as the sum scores of AVLT N1, N2, and N3.

### 2.3 Magnetic resonance imaging data acquisition

Subjects underwent magnetic resonance imaging (MRI) scans after the neuropsychological assessments. The Philips 3.0T MR system in the Affiliated Brain Hospital of Guangzhou Medical University (Philips, Achieva) was used to acquire imaging data. For each participant, an anatomical image was obtained with a sagittal T1‐weighted 3D gradient‐echo sequence (TR = 8.2 ms, TED = 3.8 ms, TI = 1100 ms, flip angle = 8°, 188 slices, slice thickness = 1 mm, gap = 0 mm, matrix = 256 × 256). Sagittal resting‐state fMRI datasets of the whole brain were obtained in 8 minutes with a single‐shot gradient‐echo planar imaging pulse sequence. The resting‐state fMRI scanning parameters wsere as follows: TE = 30 ms, TR = 2000 ms, flip angle (FA) = 90 degrees, numbers of slices = 33, slice thickness = 4 mm, matrix size = 64 × 64, and field of view (FOV) = 220 × 220 mm.

### 2.4 Image processing

Resting‐state fMRI data preprocessing was carried out using the Data Processing Assistant for Resting‐State 5.0 (DPARSF 5.0). The first ten volumes were removed to preserve steady‐state data only. The remaining images were corrected for timing differences and head motion. Subjects who had images with more than 2 mm of translational movement or more than 2 degrees of rotational movement were excluded from further analysis. The individual structural image (T1‐weighted images) was coregistered to the mean functional image after motion correction. The transformed structural images were segmented into gray matter, white matter, and cerebrospinal fluid. Nuisance signals, such as six head motion parameters, global signal, CSF signal, and WM signal were regressed out from each time series. Following this, the motion‐corrected functional images were spatially normalized into the Montreal Neurological Institute space and resampled to 3 × 3 × 3 mm^3^ using the normalization parameters estimated during unified segmentation. To reduce the effect of low‐frequency drifts and high‐frequency noise, a bandpass filter (0.01 Hz < f < 0.1 Hz) was applied for the analysis of dFC and dReho.

### 2.5 Analyses of dynamic ALFF, dynamic ReHo, and dynamic FC

The temporal variability in the spontaneous fluctuations of activity was assessed by dynamic ALFF (dALFF) and dynamic ReHo (dReHo). The Hamming window was used to slide the whole‐brain BOLD signals. A sliding window size of 100 TR and a window step of 1 TR were selected to evaluate the whole‐brain dALFF variability. By using the 100‐TR sliding window analyses, the 230 time points were segmented into 131 windows for each participant. In each window length, for a given voxel, the time series was first converted to the frequency domain using a fast Fourier transform. The square root of the power spectrum was computed and then averaged across a predefined frequency interval (0.01–0.1 Hz). The average square root was considered to be the ALFF at the given voxel.^43^ Then, the standard deviation of the ALFF values (dALFF variability) across all 131 windows was calculated to quantitatively depict the temporal dynamic characteristics of ALFF. Subsequently, we applied z standardization within the gray matter mask, and the dALFF variability maps were smoothed with a 6 mm full width at half maximum (FWHM) Gaussian kernel. ReHo reflects the degree of local regional neural activity coherence. Briefly, it was calculated as Kendall's coefficient of concordance (or Kendall's W) of the time course of a given voxel with those of its nearest neighbors (26 voxels). A sliding window size of 50 TR and a window step of 1 TR were applied to calculate the dReho variability of each voxel (181 windows),^26^, ^27^ and the other processing was the same as for the dALFF.

The temporal variability in the spontaneous fluctuations of connectivity was assessed by the dynamic FC (dFC). A previous study suggested that functional convergence of the caudal‐rostral hippocampus may be a sensitive biomarker of disease severity along the AD spectrum.^44^ Therefore, the present study selected the bilateral caudal hippocampus and bilateral rostral hippocampus as the seeds for calculating the dFC variability according to the Brainnetome Atlas (Brainnetome Atlas Viewer, vision 1.0, http://atlas.brainnetome.org/).^45^ For each sliding window, correlation maps were produced by computing the temporal correlation coefficient between the truncated time series of the seeds and all the other voxels. Consequently, 181 sliding window correlation maps were obtained for each participant. The obtained correlation maps were then converted to *z* value maps using Fisher's *r*‐to‐*z* transformation to improve the normality of the correlation distribution. Subsequently, we calculated the standard deviation of the *z* value at each voxel to assess dFC variability. Finally, we applied *z* standardization within the gray matter mask, and the dFC variability maps were smoothed with a 6 mm FWHM Gaussian kernel.^26^, ^27^

### 2.6 Statistical analyses

Demographic and clinical data were analyzed by using SPSS, version 25.0 (SPSS). The differences between the aMCI group, naMCI group, and HC group were analyzed using Analysis of Covariance (ancova), and control variables included age, sex, and years of education. The least significant difference (LSD) test was used for post hoc analyses. A chi‐squared test was used to compare the sex differences among the three groups. To examine the differences in the variability of dALFF and dReHo among the three groups, ancova was carried out to compare the group differences based on the standard deviation in the z value at each voxel within the gray matter mask, with age, sex, years of education and mean frame‐wise displacement (FD) values as control variables. The multiple comparisons of dALFF and dReHo were corrected by using Gaussian random field (GRF) theory (voxel *p* < 0.001, cluster *p* < 0.05, cluster size >10).

The one‐sample *t* test was performed to investigate the within‐group dFC variability distribution of each hippocampal seed in patients in the aMCI group, naMCI group, and HC group. The significance level was set at a *p* < 0.05 (uncorrected). To further examine the difference in dFC variability patterns among the three groups, ancova was performed on the standard deviation in the z value at each voxel within the union mask of one‐sample t test results of the three groups. Age, sex, years of education, and mean FD values were included as nuisance covariates in the comparisons. The multiple comparisons were corrected by using Gaussian random field (GRF) theory (voxel *p* < 0.001, cluster *p* < 0.05, cluster size >10).

The brain regions showing significantly different dALFF, dReHo, and dFC variability based on the results of the ancova were defined as seeds for a further post hoc analysis for comparing the groups in pairs. Partial correlations were used to investigate the correlation between the cognitive scores and the variability values of dALFF, dReHo, or dFC for each significant region, controlling for the variables age, sex, and years of education. Mediation analyses were performed to investigate the relationship between MCI subtypes (independent variable) and different cognitive scores (dependent variable), and the values of dALFF, dReHo, or dFC were regarded as mediators, with age, sex, and years of education as covariates. The mediation model was calculated in PROCESS v3.4, and the level of confidence for all confidence intervals in the output was 95% with 5000 bootstrap samples.

## 3 RESULTS

### 3.1 Demographic and cognitive information

There was one subject with naMCI, 3 subjects with aMCI, and 1 HC who were excluded because they had images with more than 2 mm of translational movement or more than 2 degrees of rotational movement. The demographic and cognitive information of the HC, naMCI, and aMCI groups is listed in Table 1. No significant difference was found in age and sex distribution among the three groups (*p* > 0.05), and the aMCI group exhibited fewer years of education than the HC and naMCI groups (*p* < 0.05). For the comparison of cognitive scores, significant differences were found in all assessments among the three groups (*p* < 0.05). In the post hoc comparisons, both the naMCI and aMCI groups exhibited worse performance in all cognitive scores, and the aMCI group exhibited lower scores in three AVLT aspects than the naMCI group (*p* < 0.05). No significant difference was found in the other assessments between the aMCI and naMCI groups (*p* > 0.05).

**TABLE 1:** Demographic data, clinical information, and cognitive function of all subjects

	HC (*n* = 74)	naMCI (*n* = 64)	aMCI (*n* = 45)	F/χ^2^ ^a^	*p*	Post hoc^b^
Male (%)	18 (27.7%)	11 (34.4%)	15 (30%)	0.574	0.751	–
Age	66.1 ± 5.0	67.6 ± 7.7	66.8 ± 8.3	0.788	0.456	–
Years of education	10.8 ± 2.9	9.0 ± 3.6	8.4 ± 3.6	8.127	<0.001**	A, B > C
Global cognition						
MMSE	27.2 ± 2.0	25.4 ± 2.4	24.6 ± 2.9	16.969	<0.001**	A > B, C
Memory						
AVLT N1‐3	21.1 ± 4.1	17.9 ± 4.3	14.5 ± 4.6	27.615	<0.001**	A > B > C
AVLT N5	6.9 ± 2.1	5.4 ± 2.2	2.3 ± 1.8	51.908	<0.001**	A > B > C
AVLT N6	6.9 ± 2.1	5.0 ± 2.5	2.8 ± 1.9	39.793	<0.001**	A > B > C
Language						
BNT	23.5 ± 2.0	19.4 ± 3.0	19.6 ± 3.6	43.931	<0.001**	A > B, C
VFT	10.4 ± 3.2	8.0 ± 2.8	8.7 ± 3.5	11.077	<0.001**	A > B, C
Executive function						
TMT B (second)	59.0 ± 19.7	83.0 ± 34.8	83.5 ± 31.9	14.861	<0.001**	A < B, C
Stroop C (second)	78.0 ± 20.6	95.3 ± 35.4	101.1 ± 38.5	8.523	<0.001**	A < B, C
Visuospatial skill						
ROCF	27.8 ± 3.8	25.7 ± 5.0	22.8 ± 7.6	11.158	<0.001**	A > B > C
CDT4	4.0 ± 0.12	3.2 ± 0.7	3.3 ± 0.9	39.974	<0.001**	A > B, C
Attention						
SMDT	35.2 ± 10.3	30.8 ± 10.5	27.2 ± 9.9	7.378	0.001*	A > B, C
DST	10.4 ± 1.9	9.0 ± 2.1	8.7 ± 2.2	10.906	<0.001**	A > B, C

Abbreviations: HC, healthy controls; aMCI, amnestic mild cognitive impairment; naMCI, nonamnestic mild cognitive impairment; AD, Alzheimer's disease; MMSE, mini‐mental state examination; AVLT N1‐3, auditory verbal learning test immediately recall; AVLT N5, auditory verbal learning test long‐term delayed recall; AVLT N6, auditory verbal learning test recognition; BNT, Boston naming test; VFT, verbal fluency test; TMT B, trail‐making test part B; Stroop C, the time of Stroop color and word test part three; ROCF, Rey‐Osterrieth complex figure test; CDT, Clock Drawing Test; SDMT, symbol digit modality test; DST, digit span test.

^a^ F refers to the two‐tailed Fisher's exact test, χ^2^ refers to the two‐tailed chi‐square test.

^b^ In post hoc multiple comparisons, A means NC group, B means naMCI group, C means aMCI group.

* Statistically significant at the 0.05 level (2‐tailed)

** Statistically significant at the 0.01 level (2‐tailed).

### 3.2 Comparison of dALFF variability

**FIGURE 1:**
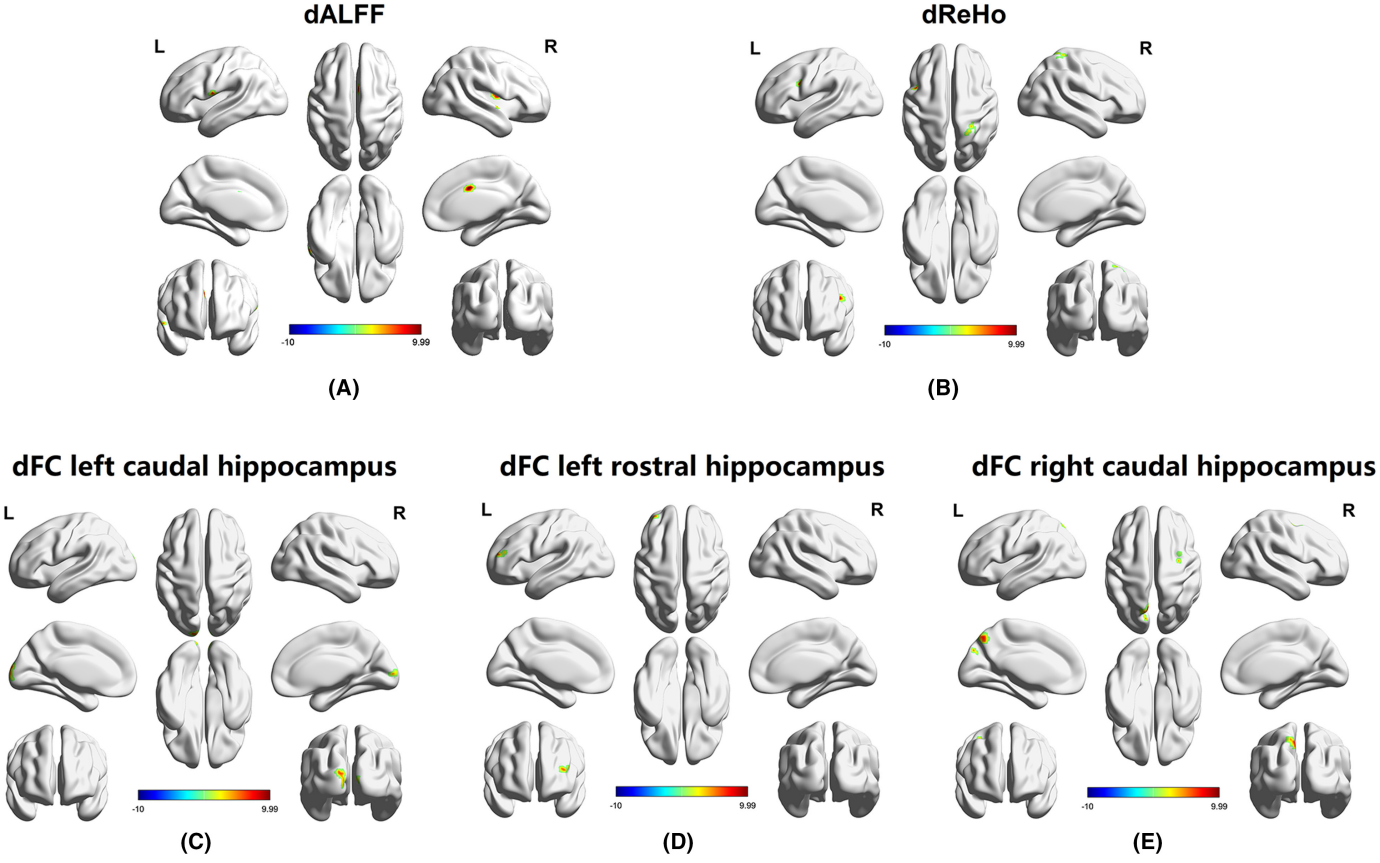
Comparison of dALFF variability, dReHo variability, and dFC variability among HC, aMCI, and naMCI groups. (A) There were significant differences of dALFF variability in left superior cerebellum, right putamen, right superior temporal gyrus, left Rolandic operculum, and right middle cingulum among the three groups. (B) There were significant differences of dReHo variability in the left inferior frontal gyrus, left precuneus, and right superior parietal lobule among the three groups. (C) There were significant differences of dFC variability between the left caudal hippocampus and left superior occipital gyrus among the three groups. (D) There were significant differences of dFC variability between the left rostral hippocampus and left middle frontal gyrus among the three groups. (E) There were significant differences of dFC variability between right caudal hippocampus and left inferior cerebellum, right caudal hippocampus and left precuneus, right caudal hippocampus and right superior frontal gyrus among the three groups. dALFF, dynamic amplitude of low‐frequency fluctuation; dReHo, dynamic regional homogeneity; dFC, dynamic functional connectivity.

**FIGURE 2:**
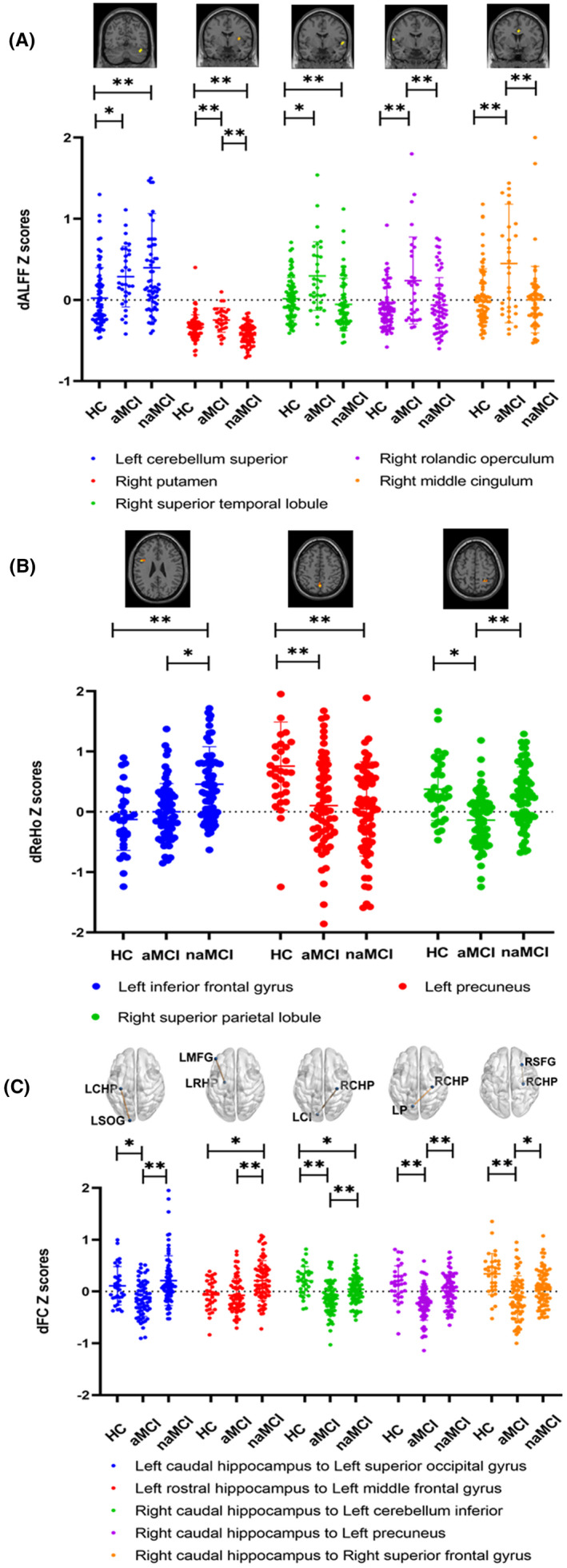
Post hoc comparison of dALFF variability, dReHo variability, and dFC variability among HC, aMCI, and naMCI groups. *p < 0.05, **p < 0.01, ***p < 0.001. dALFF variability, dynamic amplitude of low‐frequency fluctuation dReHo variability, dynamic regional homogeneity, dFC variability, dynamic functional connectivity. aMCI, amnestic mild cognitive impairment; naMCI, nonamnestic mild cognitive impairment; HC, healthy controls.

Among the HC, naMCI, and aMCI groups, there were significant differences in dALFF variability in the left superior cerebellum, right putamen, right superior temporal gyrus, left Rolandic operculum and right middle cingulum (Table 2, Figure 1A). In the post hoc comparisons, (1) both the naMCI and aMCI groups exhibited higher dALFF variability in the left superior cerebellum and right superior temporal gyrus; (2) the aMCI group exhibited higher dALFF variability in the right putamen, left Rolandic operculum, and right middle cingulum than the HC and naMCI groups; (3) compared with the HC group, the naMCI group exhibited lower dALFF variability, and the aMCI group exhibited higher dALFF variability in the right putamen (*p* < 0.05) (Figure 2A).

**TABLE 2:** Comparison of dALFF variability, dReHo variability, and dFC variability among HC, aMCI, and naMCI groups

Brain regions	Peak MNI			Cluster size	*F*
	*x*	*y*	*z*		
dALFF					
Left cerebellum superior	−39	−66	−24	12	9.51
Right putamen	33	−3	9	27	14.73
Right superior temporal gyrus	57	−3	0	18	9.75
Left Rolandic operculum	−63	−6	9	15	10.06
Right middle cingulum	3	3	33	17	11.41
dReHo					
Left inferior frontal gyrus, opercular part	−45	6	27	15	13.35
Left precuneus	0	−63	51	17	14.54
Right superior parietal lobule	36	−39	66	19	12.69
Left caudal hippocampus dFC					
Left superior occipital gyrus	−12	−99	18	64	7.69
Left rostral hippocampus dFC					
Left middle frontal gyrus	−45	45	18	47	7.42
Right caudal hippocampus dFC					
Left inferior cerebellum	−12	−84	−33	76	10.46
Left precuneus	−12	−69	48	87	11.08
Right superior frontal gyrus	30	3	63	33	11.08

### 3.3 Comparison of dReHo variability

Among the HC, naMCI, and aMCI groups, there were significant differences in dReHo variability in the left inferior frontal gyrus, left precuneus, and right superior parietal lobule (Table 2, Figure 1B). In the post hoc comparisons, (1) the naMCI group exhibited higher dReHo variability in the left inferior frontal gyrus than the naMCI and HC groups; (2) both the naMCI and aMCI groups exhibited lower dReHo variability in the left precuneus than the HC group; and (3) the aMCI group exhibited lower dReHo variability in the right superior parietal lobule than the naMCI and HC groups (*p* < 0.05) (Figure 2B).

### 3.4 Comparison of dFC variability

The results of a one‐sample t test of hippocampal dFC in HC, naMCI and aMCI were shown in Figure S1. Among the HC, naMCI, and aMCI groups, there were significant differences in dFC variability between the left caudal hippocampus and left superior occipital gyrus, left rostral hippocampus and left middle frontal gyrus, right caudal hippocampus and left inferior cerebellum, right caudal hippocampus and left precuneus, and right caudal hippocampus and right superior frontal gyrus (Table 2, Figure 1C–E). In the post hoc comparisons, (1) the aMCI group exhibited lower dFC variability between the left caudal hippocampus and left superior occipital gyrus, right caudal hippocampus and left inferior cerebellum, right caudal hippocampus and left precuneus, and right caudal hippocampus and right superior frontal gyrus than the naMCI and HC groups; and (2) the naMCI group exhibited higher dFC variability in the left rostral hippocampus and left middle frontal gyrus than the aMCI and HC groups and lower dFC variability in the right caudal hippocampus and left inferior cerebellum than the HC group (*p* < 0.05) (Figure 2C).

### 3.5 Correlation analyses

**FIGURE 3:**
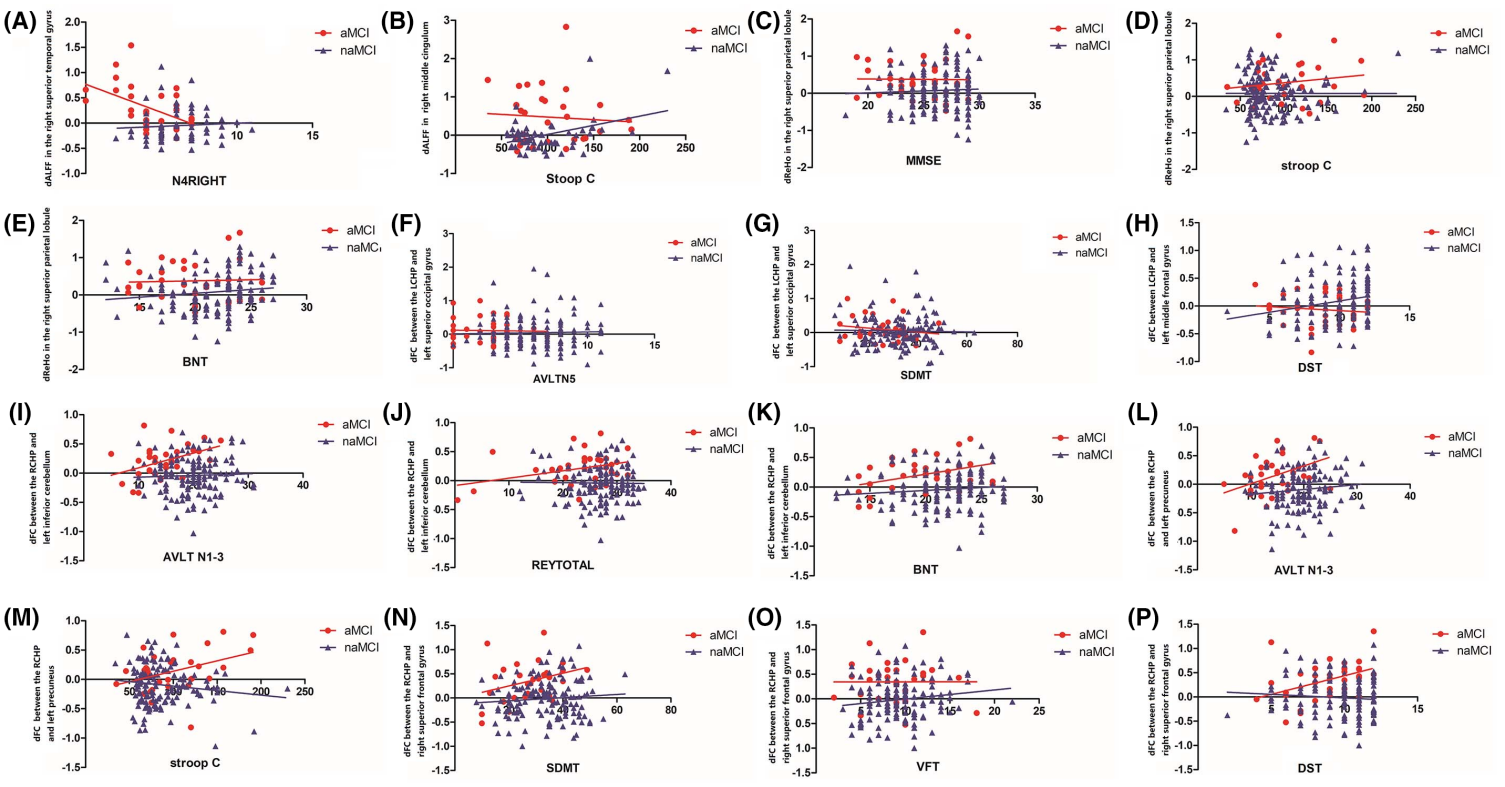
Correlations between dynamic indicators and cognitive scores in aMCI and naMCI groups. The dALFF variability in the right superior temporal gyrus was associated with AVLT N4 (*r* = −0.598, *p* = 0.002) in the aMCI group (A), and the dALFF variability in the right middle cingulum was associated with time of Stroop C (*r* = 0.464, *p* < 0.001) in the naMCI group (B). The dReHo variability in the right superior parietal lobule was associated with MMSE (*r* = −0.281, *p* = 0.028) (C), time of Stroop C (*r* = 0.411, *p* = 0.001) (D), and BNT (*r* = −0.287, *p* = 0.027) (E) in the naMCI group. The dFC variability between the left caudal hippocampus and left superior occipital gyrus was associated with AVLT N5 (*r* = −0.348, *p* = 0.007) (F) and SMDT (*r* = −0.383, *p* = 0.003) (G) in the naMCI group. The dFC variability between the left rostral hippocampus and left middle frontal gyrus was associated with DST (*r* = 0.317, *p* = 0.015) (H) in the aMCI group. The dFC variability between the right caudal hippocampus and left inferior cerebellum was associated with AVLT N1‐3 (*r* = 0.438, *p* = 0.022) (I), ROCF (*r* = 0.473, *p* = 0.013) (J), and BNT (*r* = 0.509, *p* = 0.007) (K) in the aMCI group. The dFC variability between the right caudal hippocampus and left precuneus was associated with AVLT N1‐3 (*r* = 0.508, *p* = 0.007) (L) and time of Stroop C (*r* = 0.400, *p* = 0.039) (M) in the aMCI group. The dFC variability between the right caudal hippocampus and right superior frontal gyrus was associated with SMDT (*r* = 0.474, *p* = 0.012) (N), VFT (*r* = −0.474, *p* = 0.013) (O), and DST (*r* = 0.548, *p* = 0.003) (P) in the aMCI group.

dALFF variability in the right superior temporal gyrus was associated with the AVLT N4 (*r* = −0.598, *p* = 0.002) scores in the aMCI group (Figure 3A), and dALFF variability in the right middle cingulum was associated with the time score of the Stroop C (*r* = 0.464, *p* < 0.001) in the naMCI group (Figure 3B). No other significant correlation was found between cognitive scores and other dALFF variability (*p* > 0.05). dReHo variability in the right superior parietal lobule was associated with MMSE (*r* = −0.281, *p* = 0.028) (Figure 3C), Stroop C (*r* = 0.411, *p* = 0.001) (Figure 3D) and BNT (*r* = −0.287, *p* = 0.027) (Figure 3E) scores in the naMCI group. No other significant correlation was found between cognitive scores and dReHo variability (*p* > 0.05). dFC variability between the left caudal hippocampus and left superior occipital gyrus was associated with AVLT N5 (*r* = −0.348, *p* = 0.007) (Figure 3F) and SMDT (*r* = −0.383, *p* = 0.003) (Figure 3G) scores in the naMCI group. dFC variability between the left rostral hippocampus and left middle frontal gyrus was associated with DST (*r* = 0.317, *p* = 0.015) (Figure 3H) scores in the aMCI group. dFC variability between the right caudal hippocampus and left inferior cerebellum was associated with AVLT N1‐3 (*r* = 0.438, *p* = 0.022) (Figure 3I), ROCF (*r* = 0.473, *p* = 0.013) (Figure 3J) and BNT (*r* = 0.509, *p* = 0.007) (Figure 3K) scores in the aMCI group. dFC variability between the right caudal hippocampus and left praecuneus was associated with AVLT N1‐3 (*r* = 0.508, *p* = 0.007) (Figure 3L) score and time score of the Stroop C (*r* = 0.400, *p* = 0.039) (Figure 3M) in the aMCI group. dFC variability between the right caudal hippocampus and right superior frontal gyrus was associated with SMDT (*r* = 0.474, *p* = 0.012) (Figure 3N), VFT (*r* = −0.474, *p* = 0.013) (Figure 3O) and DST (*r* = 0.548, *p* = 0.003) (Figure 3P) scores in the aMCI group. No other significant correlation was found between other dFC and cognitive scores, and there was no significant correlation between dynamic indicators and cognitive scores in the HC group (*p* > 0.05).

### 3.6 Mediation analyses

**FIGURE 4:**
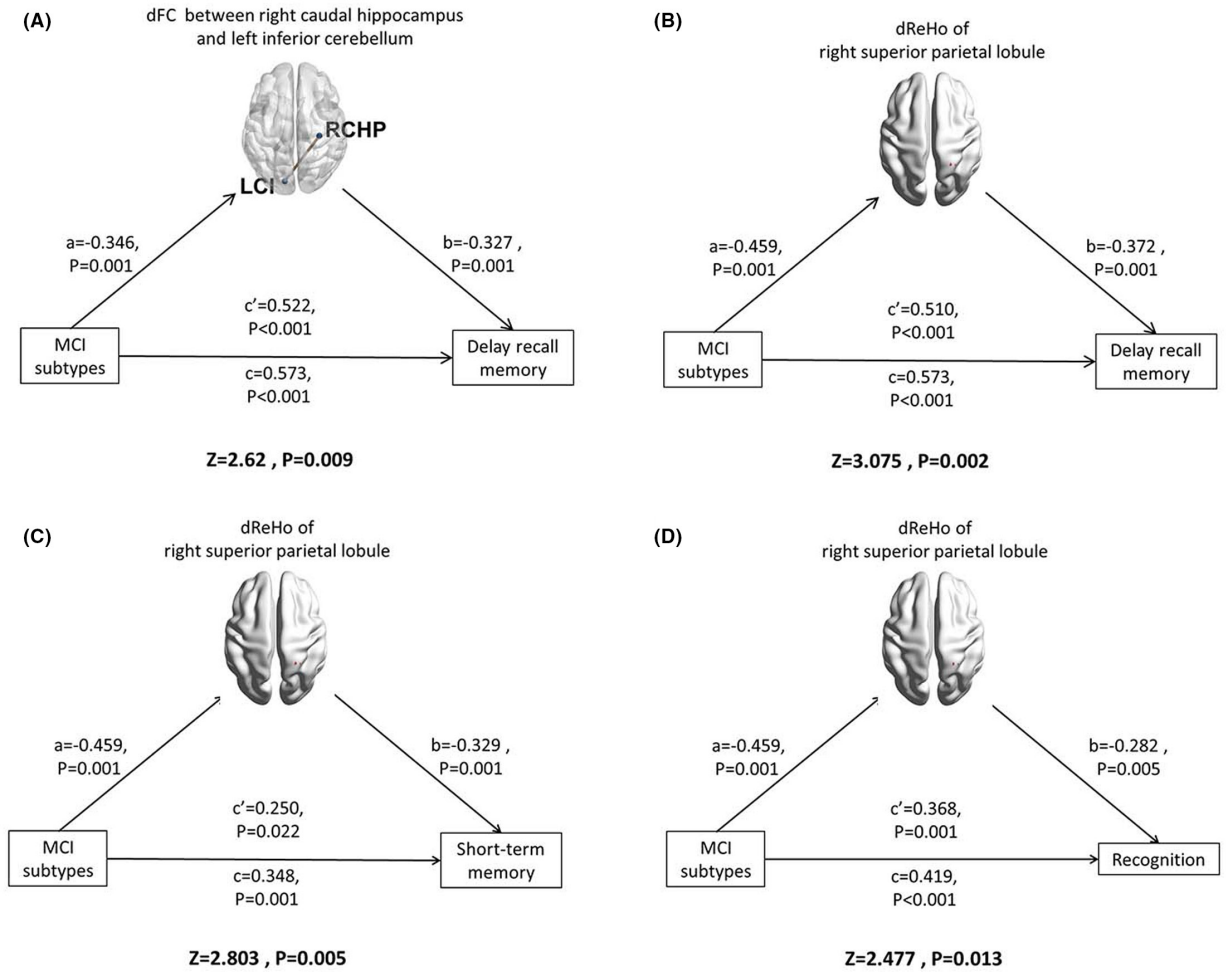
Mediated effect of dynamic brain function on the different cognitive scores between aMCI and naMCI groups. (A) The dFC variability between right caudal hippocampus and left inferior cerebellum partially mediated to the difference in delay recall memory score between aMCI and naMCI groups. (B) The dReHo variability right superior parietal lobule partially mediated the difference in delay recall memory score between aMCI and naMCI groups. (C) The dReHo variability right superior parietal lobule partially mediated the difference in short‐term memory score between aMCI and naMCI groups. (D) The dReHo variability right superior parietal lobule partially mediated the difference in recognition score between aMCI and naMCI groups.

Mediation analyses were performed with MCI subtypes as independent variables, AVLT scores (significantly different between the aMCI and naMCI groups) as dependent variables, and the dynamic values that were significantly correlated with cognitive scores as mediators. After Bonferroni correction, there were two dynamic indicators exhibiting a partially mediated effect on the differences in memory scores between the aMCI and naMCI groups, including dFC variability between the right caudal hippocampus and left inferior cerebellum for delayed recall (*Z* = 2.62, *p* = 0.009) (Figure 4A) and dReHo variability in the right superior parietal lobule for delayed recall (*Z* = 3.075, *p* = 0.002**)** (Figure 4B), short‐term memory (*Z* = 2.803, *p* = 0.005) (Figure 4C) and recognition (*Z* = 2.477, *p* = 0.013) (Figure 4D).

## 4 DISCUSSION

The present study is the first to compare the temporal variability in intrinsic brain function between aMCI, naMCI, and HC groups and provides evidence that the different functional abnormalities of some regions between aMCI and naMCI patients may only be shown in dynamic analyses but not static analyses, as demonstrated by previous studies. First, compared with the HC group, the abnormal patterns of variability of dALFF, dReHo and hippocampal dFC were different in the aMCI and naMCI groups. Second, dALFF variability, dReHo variability, and hippocampal dFC variability exhibited different associations with cognitive scores in the aMCI and naMCI groups. Third, dReHo variability in the right superior parietal lobule and dFC variability between the right caudal hippocampus and left inferior cerebellum mediated the different memory scores between the aMCI and naMCI groups.

The present study suggested that aMCI and naMCI subjects exhibited shared and specific dynamics of brain activity and connectivity. Specifically, their shared pattern included higher dALFF variability in the right superior temporal gyrus and left superior cerebellum, and decreased dReHo in the left precuneus, which were more related to abnormal activity but not connectivity. Abnormalities in the temporal gyrus, precuneus, and cerebellum in AD spectrum diseases have been repeatedly reported in previous studies.^16^, ^46^ The superior temporal gyrus plays a necessary role in spoken word recognition because it is related to auditory association and multisensory integration,^48^ and the cerebellum is crucially involved in a wide spectrum of cognitive including neurocognitive development, language function, working memory, and executive function. Therefore, the increased dALFF variability of the left superior cerebellum and right superior temporal gyrus may suggest their instability and disturbance when processing‐related cognitive tasks in both aMCI and naMCI patients. The precuneus is involved in various complex cognitive functions, such as recollection and memory, integration of information relating to the perception of the environment, cue reactivity, mental imagery strategies, and episodic memory retrieval.^47^ Hence, the decreased dReHo of precuneus may suggest its inflexible connectivity with the nearest neighboring regions when dealing with relevant cognitive information in both aMCI and naMCI patients. Overall, the present study indicated that abnormally dynamic activities of the superior temporal gyrus, cerebellum, and precuneus may contribute to the common underlying mechanism of MCI subtypes, and longitudinal studies are needed to investigate their relationship with future cognitive decline and dementia conversion.

Regarding the limited shared patterns of MCI subtypes, there were more brain regions involved in their specific patterns, and their different dynamic characteristics included not only local indicators (dALFF and dReHo) but also connected indicators (hippocampal dFC). Additionally, the associations between the dynamic characteristics and cognitive scores were different in the aMCI and naMCI groups, suggesting that cognitive impairment was related to different brain abnormalities. These results were consistent with previous opinions that the separation of MCI subtypes is not only theoretical but also backed by assessments of neuroimaging methods, neuropsychological tests, susceptible genes, and cardiovascular risk factors.^4^, ^6^, ^7^ Therefore, exploring the different dynamics of brain activity and connectivity could provide a deeper understanding of the different mechanisms of MCI subtypes and provide more therapeutic targets in preventing the conversion to neurocognitive disorders.

Among all the dynamic indicators, the superior parietal lobule seems to play the most important role in differentiating MCI subtypes because its dReHo mediated the difference in all three memory aspects (short‐term memory, delayed recall, and memory recognition) between aMCI and naMCI patients. Additionally, the dReHo of the superior parietal lobule was only significantly decreased in the aMCI group, and it was only associated with global cognition and executive function in the naMCI group, suggesting its various roles in MCI subtypes. The superior parietal lobule is involved in top‐down attention orienting, and its dysfunction causes a deficit in goal‐directed attentional orienting.^49^ Moreover, previous studies have shown that there are metabolic, structural, and functional abnormalities of the superior parietal lobule in individuals with AD, MCI, and even subjective cognitive decline,^50^, ^51^, ^52^ suggesting a close relationship between abnormalities of the superior parietal lobule and AD spectrum diseases. The present study suggested that the inflexible connectivity with the nearest neighboring regions of the superior parietal lobule may be a specific characteristic of aMCI, and it contributes to the difference in memory heterogeneity between aMCI and naMCI patients. These results are a powerful supplement to the information on the relationship between the superior parietal lobule and AD spectrum diseases, and indicate that the superior parietal lobule may be a potential target for neuromodulation in aMCI patients.

Apart from the dReHo of the superior parietal lobule, the dFC of the hippocampus was also a partial mediator of the memory heterogeneity between aMCI and naMCI patients, and their absence of abnormal dALFF and dReHo suggested that the dynamic brain dysfunction of the hippocampus was more related to connection but not activity. The hippocampus plays an important role in the cognitive map, and it is widely connected to other brain regions and involved in various complex memory processing tasks.^53^ Moreover, the hippocampus is affected early by AD pathology, and its extent of abnormalities reflects the progression of AD development.^54^ Abnormal hippocampal FC in AD spectrum diseases has been reported in many studies,^15^, ^55^, ^56^ and a recent study demonstrated that rostral‐caudal hippocampal functional convergence is reduced across the AD spectrum.^44^ Consistent with the above evidence, the present study suggested that the functional role of the rostral‐caudal hippocampus varied, and the abnormal hippocampal FC in MCI patients was static but also dynamic. These results provide a deeper understanding of hippocampal FC in MCI subtypes, and indicate that exploring the function of different subfields of the hippocampus may contribute to differentiating aMCI and naMCI.

Interestingly, the dynamic connectivity of the cerebellum also plays an important role in differentiating MCI subtypes. Specifically, the aMCI group exhibited lower dFC between the right caudal hippocampus and left inferior cerebellum than the naMCI group, suggesting the flexibility and efficiency for transporting information between these regions may reduce in aMCI patients. Additionally, the dFC between the right caudal hippocampus and left inferior cerebellum was a partial mediator of the difference in delayed recall memory between MCI subtypes. In recent years, the cognitive role of the cerebellum has attracted increasing research interest. It was reported that the cognitive cerebellum is located in lobules VI and VII in the cerebellar posterior lobe and connects to many critical nodes of the cerebral cortex, including the default mode network, hippocampus, and medial prefrontal cortex.^57^, ^58^, ^59^ Furthermore, functional abnormalities of the cerebellum have been repeatedly found in patients with AD and MCI and are involved in various cognitive processes.^30^, ^60^, ^61^ The present study confirmed that the disturbance of dynamic connectivity in the cerebellum may lead to cognitive impairment in MCI individuals, and the different patterns of abnormalities may contribute to differentiating aMCI and naMCI. The present results enhance the understanding of the role of the cerebellum in the pathophysiological mechanisms of MCI, and suggest that alterations in cortico‐cerebellar dynamic FC represent a novel approach for early differential diagnosis and a potential therapeutic target for early intervention.

Except for the mentioned brain areas, there were other regions that exhibited different patterns of activity or connectivity between the aMCI and naMCI groups, including the dALFF in the right putamen, left Rolandic operculum, and right middle cingulum, dReHo in the left inferior frontal gyrus, and dFC between the hippocampus and other regions (left superior occipital gyrus and left middle frontal gyrus). These abnormal regions were also reported in previous MCI studies,^62^, ^63^ and the present results further confirmed that the difference in functional abnormalities in MCI subtypes is widespread in the brain.^2^ Although some of these dynamic indicators were also correlated with the cognitive scores, none of them showed a mediated effect on the difference in cognitive scores between the aMCI and naMCI groups, suggesting that they may be less important than the hippocampus, cerebellum, and superior parietal lobule in differentiating MCI subtypes. Future studies applying more comprehensive neuroimaging analyses could better clarify their role in the difference in MCI subtypes.

Previous studies provided the range of the appropriate window length as 10–75 TR, step = 1 TR, and a moderate sliding window length may maximize the statistical power, because it may be an optimal balance between capturing rapidly shifting dynamic relationships (with shorter windows) and achieving reliable estimates of the correlations between regions (with longer windows).^2^, ^64^ Additionally, a sliding window size of 50 TR and a window step of 1 TR has been repeatedly used in previous studies, and they were able to capture the dynamics.^65^, ^66^, ^67^, ^68^ Therefore, the present study applied a sliding window size of 50 TR and a window step of 1 TR to calculate the dReHo and dFC. However, according to the Nyquist rule, the sampling of the low frequency 0.01 should be at least 200, and we applied a sliding window size of a window length 100 TR (200 s) to calculate the dALFF. Future studies using other window lengths could further explore the effect of sliding window size on the results of MCI patients.

There are limitations in the present study. First, the present conclusions were based on cross‐sectional analyses, and longitudinal studies are needed to further explore the associations between dynamic brain function and dementia progression in aMCI and naMCI individuals. Additionally, combining the use of CSF biomarkers and PET‐CT could clarify the relationship between temporal variability in intrinsic brain function and neurodegeneration. Second, 50‐TR window lengths were selected to measure dFC variability and dReHo variability, and 100‐TR window lengths were selected for dALFF variability analyses in the present study, but it remains unclear whether they are the best choice; this should be further explored by future studies with other window lengths. Third, the relatively imbalanced sample of aMCI and naMCI individuals may have influenced the statistical power, and the present results should be interpreted with caution. Fourth, the present study used the caudal and rostral hippocampus as the seeds for dFC variability analyses, and future studies including more seeds could provide a better picture of the pattern of dynamic connectivity in MCI individuals. Finally, the present subjects were all elderly people, and some of them had general health problems (such as hypertension, diabetes, and coronary heart disease) and were taking various relevant drugs, which may have exhibited potential confounding effects on brain function.

In summary, aMCI and naMCI patients exhibited shared and specific patterns of abnormal dynamic brain activity and connectivity. The connectivity of the hippocampus‐cerebellum and hippocampus‐frontal lobe and the activity of the superior parietal lobule contributed to the memory heterogeneity of MCI subtypes. By describing dynamic changes in intrinsic brain activity and connectivity, the present study offers a novel approach for differentiating the pathophysiological mechanisms of MCI subtypes and provides potential targets for individualized intervention.

## AUTHOR CONTRIBUTIONS

BC acquired the data, analyzed and interpreted the data, and drafted the manuscript. XZ and LH designed and conceptualized the study, analyzed and interpreted the data, and critically revised the manuscript. QW, ML, MY, MZ, HZ, ZW, XC, SZ, and GL acquired the data and critically revised the manuscript. YN critically revised the manuscript. All authors read and approved the final manuscript.

## Funding information

This study was supported by a grant from the National Natural Science Foundation of China (nos. 82101508 and 82171533), the Key Medical Specialty Construction Project of Traditional Chinese Medical Science in the 13th Five‐Year Plan of Guangdong Province, the Key Medical Specialty Construction Project of Traditional Chinese Medical Science of Guangzhou (2020–2022), the Guangzhou Municipal Psychiatric Diseases Clinical Transformation Laboratory (no. 201805010009), the Key Laboratory for Innovation Platform Plan, the Science and Technology Program of Guangzhou, China, the Science and Technology Plan Project of Guangdong Province (no. 2019B030316001). The funders had no role in the study design, data collection and analysis, decision to publish, or preparation of the manuscript.

## CONFLICT OF INTEREST

The authors have no actual or potential conflicts of interest to declare.

## Supporting information


Figure S1

## Data Availability

The data used to support the findings of this study are available from the corresponding author upon request.
