# Supplementary material for: Shared and specific dynamics of brain activity and connectivity in amnestic and nonamnestic mild cognitive impairment
Source: CNS Neurosci Ther. 2022 Aug 17;28(12):2053–65. doi: 10.1111/cns.13937 (PMC9627396; doi:10.1111/cns.13937)

Supplemental material

**Figure s1 One-sample t test of hippocampal dFC in HC, naMCI and aMCI.** LCHP, left caudal hippocampus; LRHP, left rostral hippocampus; RCHP, right caudal hippocampus; RRHP, right rostral hippocampus.


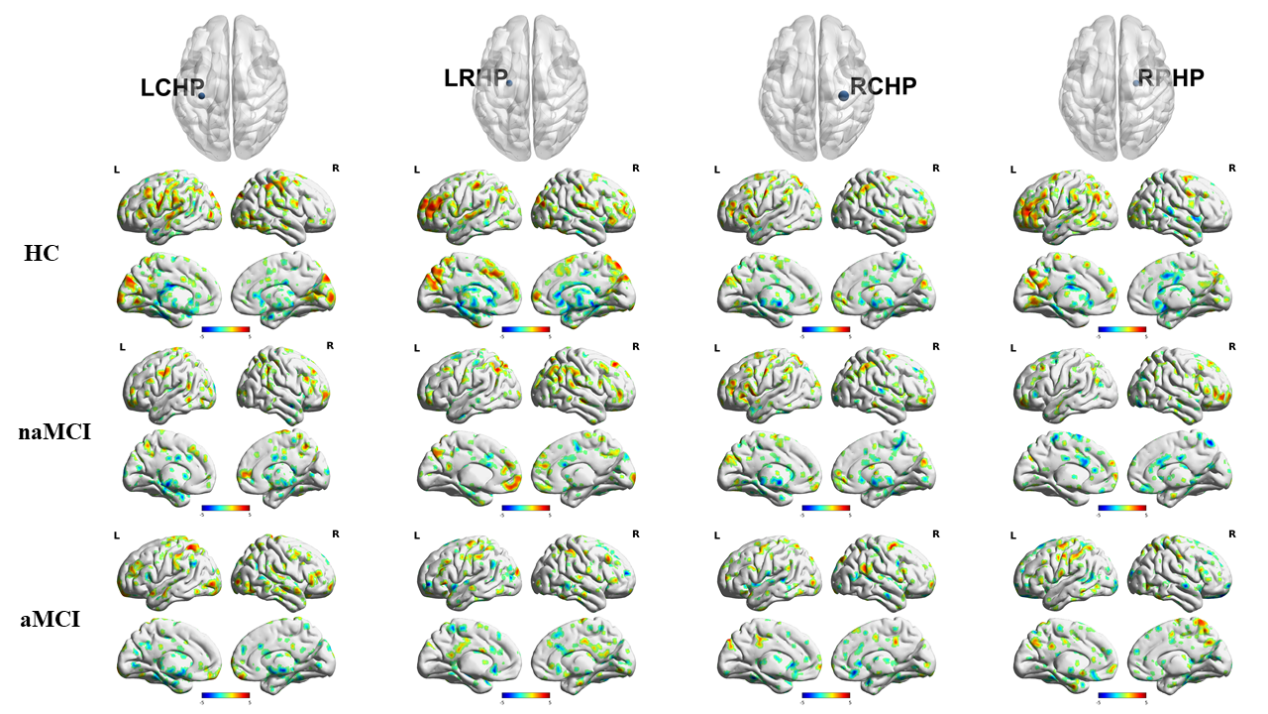

Supplement: Supplementary file 1 — Figure S1 [file CNS-28-2053-s001.docx]
